# COVID-19: Threat or Opportunity?

**DOI:** 10.17533/udea.iee.v38n3e02

**Published:** 2020-11-11

**Authors:** Mozhgan Rivaz, Seyed Mojtaba Kazemi, Mina Mosallanezhad

**Affiliations:** 1 Ph.D., Assistant professor in Nursing, Department of Nursing. Email: mrivaz@sums.ac.ir. Corresponding author. mrivaz@sums.ac.ir; 2 M.Sc in Nursing, Student research committee. Email: Mojtabakazemi9084@gmail.com Mojtabakazemi9084@gmail.com; 3 M.Sc. in Surgical Technologist, Student research committee. Email: minamo7572@gmail.com minamo7572@gmail.com; 4 School of Nursing and Midwifery, Shiraz University of Medical Sciences, Iran. Iran University of Medical Sciences Shiraz University of Medical Sciences Iran

**Keywords:** nurses, coronavirus infections, pandemics, professionalism

The COVID-19 pandemic is spreading quickly. Despite scientists’ best efforts all over the world, there is not a vaccine or definite treatment for it and the novel coronavirus remains a threat to humanity with far-reaching, and in many cases, irredeemable consequences for the economic, political, social-psychological , and cultural aspects of humans’ lives.(1)The quick transmission and fatality of the disease, absence of herd immunity, lack of or inequitable distribution of resources, e.g. Personal Protective Equipment ( PPE), and the existing challenges in the implementation of social distancing result in a daily increase in the number of victims and, consequently, an ever-expanding workload in healthcare systems worldwide. Moreover, the increasing mortality and morbidity of COVID-19 and lack of hospital beds and ventilators have led to healthcare provider’s exhaustion and burnout.(1,2) Healthcare providers, especially nurses are the most vulnerable group in the face of the current disaster. Unfortunately, In the world, many front-line experts have lost their lives.(3)

One of the indicators of social capital and the public’s risk perception is political trust. Understating a crisis, or the opposite, overstating it, lack of consistent policies or sudden changes in policies, and conflicts among administrative patterns can result in reduced risk perception and unhealthy imitation, which, in turn, can result psychological, social, and health tension in the society. The ensuing panic and anxiety pose a serious threat to public health.(4) The necessity of complying with certain prevention and control requirements, including social distancing and quarantine, have increased stress, social isolation, intra-family violence, and depression.(5)

The stigma of COVID-19 is another issue which causes individuals to delay or refuse to seek medical attention, which, in turn, results in a wider spread of the virus. COVID-19 has also disrupted monitoring programs and pediatric vaccination all over the world. According to WHO on May 22, 2020, the pediatric vaccination programs in 68 countries have been adversely affected and about 80 million babies under 1 year old have not received any primary health care, which can have serious consequences in the future.

Despite all the challenges, the COVID-19 emergency; however, it has provided opportunities which are briefly dealt with below. Solidarity in human societies following the onset of this complex crisis has underlined the essence and significance of human unity in the universe.(5) One of the most prominent themes of global collaboration is the commitment of the countries to the WHO for the production and equal distribution of a vaccine. Currently, the Solidarity Trial with the participation of 94 countries is in progress to examine the efficacy of various drugs in treating COVID-19.(6) The unprecedented crisis has created the chance for all healthcare professionals in different fields to provide comprehensive professional care by a holistic multi-disciplinary approach.

Every day, there is news about voluntary dedication of financial funds and medical equipment to hospitals, dedication of food to the lower classes, and allocation of resources to medical research, all of which are examples of “humanity in practice.” This context has been accompanied by unique opportunities and innovative solutions. Employment of technological advances, e.g. telehealth and protecting public health, to facilitate the provision of care to patients and minimize the need for face-to-face exposure is one of the interesting, effective, and economical options in preventing the spread of the virus. In many countries, the telemonitoring applications were designed to detect patients suspected coronaviruse. The rapid transmission of health information is a top priority in the control and prevention of disease. Given the problems which the pandemic has caused in the traditional ways of informing the public, distance learning, has become a necessity. Webinars on COVID-19 control and prevention and recent scientific advances in the fight against the virus are held on a daily basis all over the world.

The pandemic has underlined the significance of self-isolation facilities in residential areas. By preventing the transmission of the infection, isolation can lessen the current pressure on healthcare providers and enable them to focus on the preparation of special care units for the admission of severe cases.(7) Another apportunity to this crisis, as a result of the lockdowns and travel restrictions, is the temporary rehabilitation of the earth, especially in March and April 2020. Also, for a short time, the release of CO_2_ in China decreased to a quarter of its usual amount. In addition to reduced air pollution, there has been a significant decrease in the amount of greenhouse gases produced in all continents. Air pollutants are the major causes of respiratory system disease.(8)

Strategic planning is the analysis of basic environmental risk factors and the identification of the shortest way to achieve a goal .Under the current circumstances where our resources are inadequate and our knowledge about how to control COVID-19 is incomplete, there is an essential need for strategic planning to accomplish healthcare goals in the shortest possible time and in the most efficient way. Organizations which are constantly testing and updating their strategic plans are able to significantly reduce the undesirable consequences of the crisis and play an effective role in protecting the health and safety of their staff and patients.(9) In the International Year of the Nurse, the COVID-19 outbreak created a bold public image of nurses that saves human lives. An inhanceed public image of nurses will positively affect their self-image, professional identity, job satisfaction, and professionalism.(10) Nurses have a significant contribution to the population’s health. The current pandemic has marked a turning point in the enhancement of communication and trust between the public and the medical personnel. In most countries, millions of people have sincerely expressed their gratitude to healthcare professionals and motivated them.

Despite the fact that the COVID-19 crisis has been followed by many challenges and, with the daily increase in the number of the infected, has imposed considerable workload and expenses on healthcare systems, it has been accompanied by many opportunities, including the greater recognition of the priority of prevention over treatment in pandemics, modified life styles and improved health behaviors, unity in the world, empathetic thinking, awareness of the significance of telehealth and virtual education, recognition of the necessity of strategic planning, greater attention to the role of caregivers in promoting public health, people’s appreciation of the efforts of the healthcare providers, and innovation in making up for shortages, e.g. lack of PPE. Ultimately, the novel coronavirus has created a chance for us to reflect on our lives and gifts, including the ability to breathe without the aid of a ventilator. In addition to, the deathly crisis made further strengthened our connection to spirituality. One of the main principles in crisis management is trying to turn “threats” to “opportunities.”Thus, the invaluable experiences gained in the pandemic can help healthcare policy-makers to better plan to resolve current or future health crises.
